# Blockchain and Megatrends in Agri-Food Systems: A Multi-Source Evidence Approach †

**DOI:** 10.3390/foods15030447

**Published:** 2026-01-27

**Authors:** Christos Karkanias, Apostolos Malamakis, George F. Banias

**Affiliations:** Institute for Bio-Economy and Agri-Technology, Centre for Research and Technology-Hellas, 57001 Thessaloniki, Greece; a.malamakis@certh.gr (A.M.); g.banias@certh.gr (G.F.B.)

**Keywords:** blockchain, food systems, sustainability, megatrends, traceability, agri-food supply chains

## Abstract

Blockchain is increasingly applied in the agri-food sector to enhance traceability, data integrity, and accountability. However, its broader role in food system sustainability remains insufficiently characterized, particularly when examined against global megatrends shaping future agri-food transitions. This paper investigates how blockchain technology can reinforce sustainable, inclusive, and resilient food systems under the effect of major global megatrends. A structured literature review of peer-reviewed and industry sources was conducted to identify evidence on blockchain-enabled improvements in transparency, certification, and supply chain coordination. Complementary analysis of a curated dataset of European and international pilot implementations evaluated technological architectures, governance models, and demonstrated performance outcomes. Additionally, stakeholder-based foresight activities and scenarios representing alternative blockchain adoption pathways, developed within the TRUSTyFOOD project (GA: 101060534), were used to examine the interconnection between blockchain adoption and megatrends. Evidence from the literature and pilot cases indicates that blockchain can strengthen product-level traceability and improve verification of sustainability and safety claims. Cross-case analysis also reveals persistent constraints, including heterogeneous technical standards, limited interoperability, high deployment costs for smallholders, and governance risks arising from consortium-led platforms. Blockchain can function as an enabling digital layer for sustainable and resilient food systems and should be embedded in wider, participatory strategies that align digital innovation with long-term sustainability and equity goals in the agri-food sector.

## 1. Introduction

The revolution of global food systems is a key initiative to confront crucial challenges, including climate change, food insecurity, environmental degradation, and socioeconomic disparities [1,2]. Food systems face significant pressures from megatrends like demographic shifts, resource scarcity, urbanization, and shifting consumer preferences [3]. To overcome these challenges, coordinated, multi-level actions based on technological and policy innovations are required [4].

In this context, blockchain technology (BCT) has emerged as a widely applied digital tool to support sustainable food systems. BCT supports secure, immutable, and transparent data recording, which is fundamental for safeguarding traceability and accountability within the agri-food supply chain [5,6]. This process addresses long-standing issues, including food fraud, counterfeiting, and traceability gaps, thereby supporting compliance with sustainability standards [7,8].

During the last decade, BCT applications in agriculture and food systems have flourished, incorporating traceability, contract management, certification, as well as real-time monitoring [9,10]. For instance, noteworthy case studies like Walmart’s blockchain-enabled pork supply chain in China have showed BCT’s potential for improving efficiency and product safety [11,12]. Nevertheless, the application and scalability of BCT face multiple challenges, including fragmented infrastructure, excessive operational costs, regulatory uncertainty, and the risk of expanding digital divides [13,14].

The above-mentioned technological innovations are deeply related to several megatrends, such as digitalization, urbanization, environmental change, as well as evolving societal values [3]. The achievement of innovation in food systems is significantly supported by the interaction of these trends, which can enable efficient, inclusive, and resilient food production and supply [4]. On the other hand, the lack of sufficient governance and stakeholder engagement risk missing opportunities for real change or causing harmful effects, including technology-driven exclusion or food safety issues [12,15].

Furthermore, foresight activities and scenario analyses repeatedly underline the importance of incorporating BCT within food systems to address the related challenges and opportunities caused by global megatrends [2]. For example, blockchain can enable collaborative certification schemes, carbon footprint tracking, and adaptive food governance, given that policies strengthen interoperability, inclusivity, and data integrity [6,16].

Blockchain appeared to considerably support food systems to meet sustainability and resilience, yet its transformative capability will be broadly shown only through coordinated effort across technology and governance [17]. This study investigates how BCT’s capabilities can be advantageously aligned with global megatrends to provide adaptive, inclusive, and sustainable food systems.

Regardless of rapidly expanding research on blockchain in agri-food systems, the majority of the reviews predominantly focus on technical architectures, traceability performance, and implementation barriers in specific value chains. These studies offer valuable visions of how BCT can improve data integrity and supply chain transparency, yet they infrequently position blockchain within the broader dynamics of global food system transformation or systematically link it to cross-cutting megatrends like digitalization, climate change, and evolving consumer values. This study advances the literature by following a multi-source evidence approach that mixes a structured review of peer-reviewed and grey literature, an empirical database of European and international pilot implementations, as well as stakeholder-based foresight scenarios. This design enables an integrated assessment of how blockchain can support sustainability, resilience, and inclusion in agri-food systems when viewed through the lens of major global megatrends. Building on this gap, the study aims to (i) identify key application areas of BCT in agri-food systems and their established outcomes, (ii) characterize critical technical, organizational, and governance barriers and enablers of adoption, and (iii) examine how blockchain interconnects with selected global megatrends based on both current evidence and forward-looking scenarios.

## 2. Materials and Methods

### 2.1. Scope

The main objective of this research is to provide a thorough assessment of the evolving role of blockchain technology in shaping the sustainability, resilience, and transparency of food systems compared to the pervasive global megatrends. Modern food systems face a unique set of challenges, such as accelerated urbanization, climate change, the cumulative demand for ethically sourced products, and the requirement for advanced technological incorporation [18]. To understand these dynamics, a thorough perception of the interchange between structural, technological, and societal drivers, which define both existing and future food production and distribution landscapes, is required [19].

This study focuses on evaluating how blockchain, as a digital tool, can provide attractive solutions to the challenges faced by several modern food supply chains. Blockchain’s critical features include decentralized record-keeping, traceability, and immutability. In this context, BCT directly addresses common food sector issues, including transparency deficits, fraud, biased market practices, and traceability inadequacies, that have long undermined consumer trust worldwide [20,21]. BCT application in this sector ensures extensive improvements both in quality control and monitoring of sustainability certificates through the supply chain [19].

In addition, this paper highlights the context-dependent nature of BCT implementation investigating the background requirements needed for its successful adoption in agri-food systems. Key factors, including the prevailing regulatory environment, market readiness, and digital literacy, are examined in depth [22]. It is also identified that for BCT to bring sustainable value, it must be integrated into systems of good governance, capacity building, and multi-stakeholder engagement. On the other hand, current literature underlines the risk of a wide technology push without special attention to these enabling environments, which may lead to digital exclusion or the initiation of new weaknesses [23].

In this framework, this paper combines findings from recent reviews, scenario models, and policy-focused analyses from both academic institutions and international organizations. Furthermore, incorporating results from experimental studies, this research addresses how blockchain-driven traceability and product certification can provide pathways to achieve sustainable consumption and resource management [20,21].

Additionally, this paper highlights the importance of overcoming inertia, which have historically hindered agri-food system adaptation [22,24]. By critically assessing the impacts of megatrends, including demographic shifts, digitalization, and climate change, the consultation intends to disclose insights for researchers, practitioners, and policymakers positioning themselves at the forefront of food system innovation [18]. Finally, the research seeks to provide strategic guidance on how BCT can be controlled to promote sustainable, transparent, and future-ready food systems.

### 2.2. Methodology

**Figure 1:**
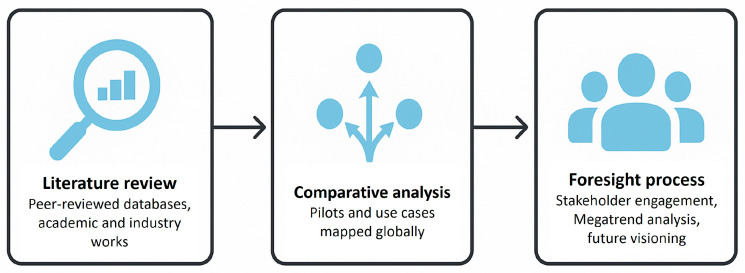
Methodological framework.

This research follows a consolidated, multi-step methodological approach to investigating blockchain’s contribution potential in the agri-food sector. This study adopts a mixed-method design combining three complementary evidence sources: (i) a structured literature review, (ii) a comparative analysis of European and international blockchain pilot deployments in agri-food systems, and (iii) a stakeholder-driven foresight process to construct future adoption scenarios (Figure 1). In addition, the analysis integrates global megatrends as a contextual framework to assess the broader drivers influencing food system transformation. The proposed approach allows both empirical assessment of current blockchain applications and forward-looking exploration of alternative development pathways.

To ensure analytical consistency across the three components, the study follows a triangulation logic in which each evidence stream contributes a distinct but complementary perspective. The structured literature review provides scale by integrating patterns of reported applications, benefits, and risks across diverse agri-food contexts. Furthermore, the pilot and use-case database adds empirical depth by examining real-world implementation characteristics, including technological architectures, governance arrangements, and performance outcomes. The stakeholder-driven foresight scenarios extend the analysis into feasible futures, allowing assessment of how megatrends and alternative governance logics may shape blockchain deployment pathways. Rather than allocating formal quantitative weights to each stream, the analysis uses the literature as a baseline, tests and enhances these insights through the pilot cases, and then interprets the credibility and distributional implications of different futures through the scenarios.

#### 2.2.1. Structured Literature

**Figure 2:**
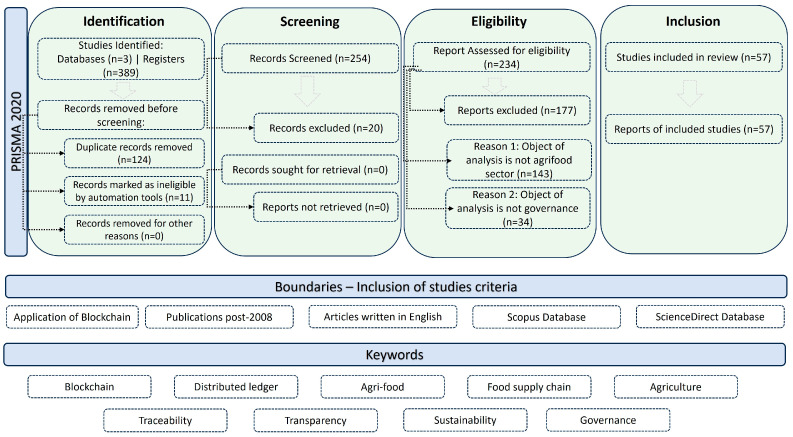
Study retrieval process [26].

The study originated with a structured literature review, to investigate the current state and main barriers of blockchain adoption in the agri-food sector (Figure 2). The review was conducted through peer-reviewed databases developed in the context of blockchain applications in food traceability, trusted recording, sustainability, and digital transformation. Data sources included academic and industry works to depict technological, regulatory, and market perspectives, as well as digital maturity and readiness of the agri-food market. The review highlighted existing barriers, enablers, and thematic research gaps.

A structured literature search was conducted to identify peer-reviewed and authoritative sources examining blockchain applications in the agri-food sector. Searches were performed in Scopus and ScienceDirect, while Google Scholar was used to capture relevant grey literature. Grey literature is defined in this study as non-commercial, policy- or practice-oriented documents, such as European Union reports, white papers, project deliverables, and technical briefs produced by international organizations or industry consortia. Grey literature sources were included only if they (i) addressed blockchain applications in agri-food or food supply chains, (ii) were issued by recognized institutions or multi-stakeholder initiatives, and (iii) provided at least a basic description of methods, system design, or implementation context. The search covered the period 2008–2025 and employed the following Boolean query: (“blockchain” OR “distributed ledger”) AND (“agri-food” OR “food supply chain” OR “agriculture”) AND (“traceability” OR “transparency” OR “sustainability” OR “governance”).

Studies were eligible for inclusion if they (i) were peer-reviewed articles, conference papers, or institutional reports, (ii) focused on blockchain applications in agricultural, food, or traceability contexts, and (iii) reported empirical findings, pilot evaluations, or conceptual frameworks. Exclusion criteria comprised (i) publications focused solely on cryptocurrency or financial services, (ii) non-English documents, and (iii) studies lacking methodological transparency.

Screening followed the latest version of the Preferred Reporting Items for Systematic Reviews and Meta-Analyses (PRISMA) protocol [25,26]. The initial database search yielded 389 records, which were imported into a reference manager and screened following the latest PRISMA guidelines. After removal of duplicate records, 254 unique publications remained for title and abstract screening, of which 20 were excluded as out of scope (e.g., cryptocurrency-focused, non-agri-food applications, or lacking methodological detail). After removal of duplicate records, 234 unique publications were assessed against the inclusion and exclusion criteria, leading to the exclusion of 177 studies mainly because they did not report blockchain applications in agri-food or did not provide sufficient information on system design or outcomes. In total, 57 studies were retained for detailed analysis. For each included study, we coded a standard set of variables, including (i) agrifood subsector and value-chain stage, (ii) geographical scope, (iii) blockchain type (public, private, or consortium) and consensus mechanism, (iv) integration with external data sources, such as IoT devices, certification schemes, or oracles, (v) reported benefits and performance indicators (e.g., recall time, audit effort, and data granularity), and (vi) barriers and enabling factors related to technology, regulation, organization, and governance.

Complementing this research, findings from desk research, surveys, and interviews with industry stakeholders were also utilized [27]. These findings provided insights concerning good practices, technical and regulatory barriers, digital maturity, and the drivers of blockchain adoption, focusing mainly on technology’s alignment with data integrity, traceability, and trust. Additionally, digital skills gaps and limitations related to costs, technical complexity, and regulatory uncertainty were identified.

#### 2.2.2. Pilot and Use-Case Database Analysis

To complement the literature findings with practice-based insights, this study developed a comprehensive database of blockchain pilot projects and use cases in the agri-food sector. These cases were identified through multiple sources, including EU project repositories (CORDIS and EIP-AGRI), peer-reviewed publications, industry white papers, and the internal pilot survey conducted within the TRUSTyFOOD project [27]. The final dataset comprised 147 blockchain-enabled agri-food pilots implemented across the 24 EU Member States and 7 associated countries in the European area, North Africa, Middle East, America, Asia, and Oceania, thereby providing a diverse representation of technological maturity, governance approaches, and value-chain configurations.

Case selection followed a goal-directed sampling strategy designed to capture diversity in product type, value-chain segment, governance model, and geographical coverage. Projects were only included if they (i) implemented blockchain or distributed ledger solutions in the agri-food supply chain, (ii) reported adequate information on system architecture, actors, and objectives, and (iii) provided some indication of performance outcomes. The resulting database includes a higher concentration of European initiatives and pilots, reflecting the availability of detailed documentation and English-language sources. This introduces potential regional and sectoral biases that are explicitly acknowledged in the analysis, and the findings should, therefore, be interpreted as indicative of current leading-edge experimentation rather than a fully global register of blockchain applications.

Pilots were analyzed using qualitative comparison to identify recurring technological choices, governance structures, and demonstrated benefits. A cross-case synthesis approach was applied to map patterns, evaluate consistency across implementation contexts, and triangulate the findings with evidence extracted from the structured literature review. This combined assessment provided a robust empirical basis for understanding how blockchain solutions are currently deployed in food systems and which conditions influence their performance and scalability.

Across the pilot cases, several distinct data-assurance mechanisms were identified. These include (i) device-based assurance, where IoT sensors automatically capture and upload measurements (e.g., temperature and location) to the ledger; (ii) certification-based assurance, where third-party audits or scheme certificates are recorded on-chain; (iii) process-based assurance, where standard operating procedures and workflow events are logged as transactions; (iv) oracle-based assurance, where external data feeds (e.g., market prices and satellite indices) are integrated via trusted intermediaries. Finally, the methodology integrated results from a stakeholder engagement plan implemented in the context of the TRUSTyFOOD project, as described by Kingfisher et al. (2025) [28]. The participatory process involved a series of scenario-based workshops and visioning sessions with a varied group of stakeholders, including farmers, producers, regulators, digital technology providers, policymakers, NGOs, and consumer representatives. Within this process, shared long-term visions, current barriers, and actionable roadmaps to transition toward sustainable, BCT-enabled food systems were identified.

#### 2.2.3. Stakeholder Engagement and Scenario Development

The development of future food system scenarios was supported through a structured, multi-actor engagement process implemented within the TRUSTyFOOD project between 2023 and 2024. Stakeholder participation in workshops and sectoral working groups was designed to ensure representation across the agri-food value chain, involving five key groups of farmers and their organizations, agri-food companies (processors and retailers), societal organizations and non-governmental organizations, blockchain experts (companies, researchers, investors, and innovators), as well as policymakers and regulators. Participants were selected using a purposive sampling strategy to capture diverse expertise and ensure that scenario outputs reflect real-world needs, constraints, and opportunities across the food system [27,29].

The study realizes that stakeholders see blockchain-enabled transparency in agri-food supply chains as both valuable and challenging. Supply chain visibility is linked to improved traceability, food safety, and trust, but, on the other hand, raises concerns in terms of competitive secrecy, data overload, and unequal exposure of information. Perceptions differ among actors who benefit from openness and those prioritizing privacy, making transparency a tool that can both enable and hinder blockchain adoption in this sector [29].

The varied representation through the stakeholders’ engagement process safeguarded that the scenarios were developed based on real-life opportunities and challenges faced by participants from farm to table and policy to market. Through structured dialogue and collective foresight, the stakeholders explored specific megatrends, uncertainties, and value-driven pressures that affect the future of food systems. Accordingly, three typical scenarios have been developed, each proposing a separate pathway for the incorporation of technology, sustainability, and governance in the agri-food sector [28,29].

##### Scenario 1: Efficiency, Transparency, and Resilience

Scenario 1 depicts a food system in which digitalization is predominantly driven by large agri-food corporations, technology providers, and global retailers. In this scenario, blockchain is deployed primarily to optimize logistics, enhance supply chain reliability, and strengthen risk management. Integration with IoT, data analytics, and automated quality monitoring enables real-time traceability and improved operational efficiency. Sustainability objectives are met primarily through improvements in supply chain management and resource use efficiency, though risks of exclusion persist for small-scale producers and regions with lower digital capacity. Although consumer trust in the food system is strengthened by data integrity, the advantages tend to focus on capitalized actors [28].

##### Scenario 2: Compliance and Accountability

In Scenario 2, public authorities play a central role in shaping digitalization trajectories. Blockchain functions as a regulatory infrastructure supporting mandatory traceability, certification, environmental reporting, and compliance monitoring. National authorities adopt substantial strategies grounded on community needs for environmental stewardship, food safety, and fair access. Digital technologies, like blockchain, are mainly employed as regulatory pillars, facilitating transparent data flows, certification schemes, and monitoring for compliance with regulatory standards [30]. Scenario 2 underlines inclusiveness via standardized protocols, incentives for digital adoption, and support for smallholder involvement. Compliance and traceability are improved, but challenges also arise in terms of administrative perplexity and the requirement for coordination among public and private stakeholders.

##### Scenario 3: Ecologically Integrated and Place-Based

Scenario 3 reflects a decentralized, community-driven adoption pathway in which blockchain is implemented to strengthen agroecological practices, local food networks, and cooperative governance structures. In this context, digital, decentralized technologies are employed to advance agroecological methods, circular economies, and improved producer–consumer relationships. Complementary certification and data governance mechanisms support supply chain visibility and trust at a local level. Furthermore, greater autonomy is achieved through the democratization of digital infrastructure, and resilience is enhanced by promoting place-based models allied with ecological and social values. Nevertheless, issues of scalability, interoperability, and fair access to facilitating technologies remain.

### 2.3. Megatrends: Forces Shaping Global Food Systems

Megatrends are considered key drivers that play a critical role in shaping the evolution of societies and economies [31]. More specifically, in food and agriculture, these megatrends incorporate technological innovation, shifting demographics, climate action, resource scarcity, evolving consumption, and new governance models. Systematic reviews highlight that realizing these trends is fundamental for efficient strategic foresight, adaptive policy design, and resilience developing [32].

The European Commission’s Megatrends Assessment Tool highlights fourteen key megatrends, such as technological acceleration, climate change, urbanization, inequalities, changing labor and migration, as well as health challenges [33]. These megatrends are interactive technological advances (e.g., enable smarter supply chains and precision farming), while digital platforms and AI transform trade and consumption habits [8]. In parallel, climate change and resource scarcity reflect the need for sustainable agriculture and efficient water and land use.

Explanatory workshops conducted by the JRC and other EU bodies have revealed the significance of engaging stakeholders from across disciplines and geographies, which can help record the relevance and interconnections of megatrends, detect potential risks, and rank advanced responses [27]. Furthermore, latest reports emphasize that resilience in food systems will increasingly rely on integrating smart technology, fitting business models to demographic shifts, and facilitating fair access through policy innovation [4].

Notably, megatrends rarely act separately. Their interaction develops both challenges, including converging resource scarcity and health shocks, and opportunities for collaborations in sustainability, digitalization, and inclusion [8,31]. Realizing and leveraging the complexity of megatrends is critical for guiding transformation in agriculture and food policy—from scenario planning and research investment to designing robust regulatory and market structures [32].

## 3. Results

### 3.1. Blockchain as an Enabler of Agri-Food Sector Transformation

Blockchain technology has been identified as a key driver in the emerging digital transformation of food systems, offering a rapid solution to persistent issues in trust, information accuracy, and transparency within value chains. Based on its original purpose as a dispersed digital archive maintained by a network, blockchain primarily redefines how transaction data are validated and stored, and safeguarding records are tamper-resistant and widely accessible [34,35].

Blockchain was consistently shown to enhance traceability precision and strengthen data integrity within agri-food supply chains. Immutable and tamper-evident ledgers improve the reliability of product information, thereby reinforcing consumer trust and facilitating regulatory compliance. Evidence from organic and fair-trade value chains further demonstrates that certification verification becomes more accurate and fraud is reduced when blockchain is combined with digital identifiers, such as QR or RFID tags and IoT-based monitoring systems [35]. Additionally, several studies highlight that real-time or near-real-time tracking capabilities enabled by blockchain substantially improve food safety responsiveness and recall management in complex, multi-actor supply chains. In general, improvements in traceability performance represent the most consistently reported benefit of blockchain adoption, with numerous implementations documenting reduced audit times, enhanced anomaly detection, and more reliable end-to-end visibility across the supply chain.

Benefits identified across the literature and pilot cases include shorter audit preparation times, faster identification and recall of affected batches, finer-grained information on product histories, and reduced manual paperwork for supply chain actors. Additional reported outcomes are higher perceived trust among consumers and stakeholders exposed to blockchain-enabled product information, as well as more robust support for environmental and social claims through linkage with recognized certification schemes and verifiable indicators (Table 1).

A key characteristic of BCT is decentralization. Contrasting conventional record systems that rely on centralized control, BCT allows decision-making among several network members. This can increase data sovereignty, support equal access, and decrease reliance on intermediaries. Nevertheless, the degree of decentralization is not consistent [36]. Open, public blockchains enable universal participation, while other private or consortium chains engage selected actors and governed access rules. This balance between open and closed systems addresses significant considerations concerning inclusion, control, and the risk of marginalizing less influential participants, including small producers or local enterprises [37].

The structured literature analysis emphasized blockchain’s capacity to enhance transparency across agri-food supply chains by enabling shared, verifiable access to distributed records. This increased visibility can reduce information asymmetries and strengthen accountability; however, several studies highlight that blockchain does not eliminate the need for trust but rather restructures it. Instead of relying on a central authority, users depend on the correct operation of the distributed network and on the behavior of validators, miners, or consortium members, depending on the blockchain type. Blockchain records are open to be viewed by various authorized parties, creating verifiable, permanent logs of transactions or product histories. In terms of the food sector, this transparency emphasizes efforts in food safety, anti-fraud measures, and compliance with sustainability certifications [38]. In parallel, there may be legitimate concerns regarding sensitive information being made too extensively available, which may bring challenges for privacy and competition [39].

At the same time, traceability safeguards that the whole product’s process from origin to consumer is accurately monitored and easily retrievable. This reinforces trust in food origin, facilitating prompt and targeted responses to food safety incidents, and supporting sustainability reporting, including certification of organic production or carbon tracing [40]. Nevertheless, efficient end-to-end traceability requires the incorporation of additional tracking technologies and robust data input systems [41,42].

In permissioned and consortium systems—common in agricultural applications—governance arrangements may inadvertently centralize power among dominant actors, potentially limiting smaller producers’ influence over data-sharing rules. Moreover, the literature notes that data integrity is constrained by the quality of incoming data, since blockchain systems remain vulnerable to inaccurate or manipulated off-chain information, a challenge widely referred to as the oracle problem. Data integrity is applied on blockchains through cryptographic links among records, making any unauthorized modification immediately evident. This facilitates reliable regulatory reporting and substantial digital audits by certifying that historical information has not been tampered with. However, the quality of blockchain’s assurance relies on the utilization of accurate, reliable data [43].

Finally, the creation of digital tokens to represent assets or certifications facilitates new forms of economic and environmental incentive mechanisms. Within food systems, tokenization can support trading of carbon credits, proof of origin certificates, or even access to loyalty and sustainability rewards. While increasingly used, this potential must be carefully managed to avoid deliberate impacts like market speculation or insufficient oversight of claims [44].

Regardless of these opportunities, BCT presents considerable risks and limitations. There is a growing concern of excluding smallholder farmers from technology, especially in low-income regions, because of lack of access, digital skills, or infrastructure. Additionally, blockchain has relatively high energy and resource demands, especially with public, proof-of-work-based records, thus sustainability and environmental questions arise. Moreover, new governance and liability gaps can emerge in the case where blockchain groups are controlled by powerful actors, which can potentially deteriorate decentralization goals. Ultimately, uneven technological standards and lack of interoperability between platforms are still considered major challenges, restraining the wider adoption of BCT in global food systems [45].

### 3.2. Megatrends Relevant to the Food System and Blockchain

The agri-food sector is experiencing intense transformations shaped mainly by several global megatrends. These long-term structural changes are reshaping how food is produced, treated, transported, and consumed, while they are affecting the challenges and opportunities for technological innovation within the food supply chain. BCT appears to be a distinctive, enabling infrastructure specifically due to its capabilities, which align profoundly with the priorities set by these key megatrends. In this section, the interaction between six megatrends is explored, including those of climate change and environmental degradation, growing consumption, aggravating resource scarcity, accelerating technological change and hyperconnectivity, demographic imbalances, as well as shifting health challenges. The current and prospective roles for blockchain in providing solutions to their impact within agri-food systems are also investigated.

#### 3.2.1. Climate Change and Environmental Degradation

Across all evidence streams, climate-related pressures emerged as the most influential megatrend. Literature and pilots frequently implemented blockchain for monitoring environmental indicators, such as fertilizer use, irrigation practices, carbon emissions, and biodiversity impacts. Climate change, along with continuing environmental degradation, remains one of the most consequential megatrends for agriculture and food systems. Food production represents one of the main sources of global greenhouse gas emissions, land conversion, as well as biodiversity loss. Rising temperatures increased the occurrence of severe weather events and thus shifted public and regulatory awareness to sustainability demands and new innovative tools for apparent monitoring, adaptation, and verification [32].

Blockchain technology addresses most of these needs by supporting immutable and transparent monitoring systems across food supply chains. For instance, BCT enables the certification of sustainable farming practices, from reduced fertilizer and pesticide use to agroecological management. By recording crucial environmental data, including water usage, carbon footprints, and biodiversity metrics, blockchain allows all interested parties both to validate environmental arguments and adjust to changing regulations, standards, or market incentives. In addition, this supports new schemes of carbon markets, credits for ecosystem services, and transparent mechanisms for reporting progress toward climate and circular economy goals [46].

Blockchain can also empower rapid and trusted responses to environmental crises like droughts, crop catastrophes, or food safety recalls following contamination by ensuring that each actor in the supply chain has simultaneous access to accurate, detailed information. Food system actors and regulators continuously expect end-to-end traceability of inputs. In this context, blockchain is considered a backbone for developing climate-resilient and environmentally accountable food production systems [47].

#### 3.2.2. Growing Consumption

Global food consumption is uninterruptedly increasing due to demographic growth, urbanization, and changing dietary habits, particularly in emerging markets facing a global shift toward more varied and resource-intensive diets. Blockchain traceability tools provide transparency to verify the integrity of sustainability, nutrition, and ethical sourcing claims, which are crucial in globalized and varied food markets. This ability facilitates consumers to make informed and responsible choices, promoting the increasing demand for sustainable, ethically sourced, low-impact, and circular products [48].

Additionally, BCT supports supply chain actors, including producers and retailers, to adopt data-driven systems that directly associate supply with demand, thus limiting gaps that result in overproduction, food losses, and food waste. Both real-time tracking and digital recording of inventory flows, accompanied with IoT sensors, support monitoring of quality life, organize recalls, and fast-track distribution of remaining goods, eliminating their environmental and financial impacts.

The transparency and granularity provided by BCT further promote product variation and personalization. Blockchain affiliates food systems with the increasing volume of consumption but also with the ascending expectations for quality, ethics, safety, and sustainability among varied community groups. Rising consumption volumes and shifting dietary preferences were consistently linked to increased demand for product transparency. Literature and pilot cases showed strong uptake of blockchain for provenance verification and sustainability claims, while stakeholders emphasized transparency as a key driver of consumer trust. Several pilots demonstrated the use of blockchain to reduce food loss through better demand forecasting and inventory visibility supported by IoT integrations [49].

#### 3.2.3. Aggravating Resource Scarcity

Modern agriculture and food production rely significantly on specific natural resources, including land, water, and energy. The unbalanced utilization of these resources depicts resource scarcity as one of the most critical risks to food security. Blockchain can facilitate the rational management of these scarcities at multiple levels by providing a set of digital tools. Smart contracts can also support compliance with water-sharing agreements or certification standards (e.g., organic production and low water-use crops). Blockchain records also support efficient, tamper-proof documentation of the amount of water, fertilizer, or energy used per crop or per season, enabling accurate benchmarking [50,51].

In parallel, BCT supports market-based mechanisms rewarding conservation, including tradable water rights or compensations for reduced inputs. Moreover, blockchain can enable access to premium markets, finance, and innovative solutions for producers by offering a transparent, standardized, and auditable system for reporting on resource footprints [52]. This helps in strengthening regulatory acceptance for resource-efficient practices, facilitating the transition to circular agri-food systems.

#### 3.2.4. Accelerating Technological Change and Hyperconnectivity

Digitalization and technological progress, including advanced analytics, Internet of things, robotics, and blockchain, are transforming food systems and agriculture. Hyperconnectivity creates opportunities for increased responsiveness, inclusiveness, and transparency, yet brings additional vulnerability and complexity. When associated with other Industry 4.0 technologies, BCT facilitates automated, real-time monitoring and management of the whole food supply chain. For instance, smart contracts can automatically activate payments, renew certifications, or restrict access to users with verified data only, like certified farmers [53]. Most importantly, hyperconnectivity provides smallholders and startups with access to global digital markets, promoting digital inclusion and system interoperability.

In addition, hyperconnected blockchain schemes promote verified food experiences, while making supply chains more resilient to cyberattacks, fraud, and logistics disruptions. At a policy level, blockchain supports regulatory compliance and decentralized food safety monitoring, accelerating the capacity of food systems to respond quickly to market, environmental, or societal shocks [53,54].

#### 3.2.5. Demographic Imbalances

Demographic imbalances, such as aging populations in developed regions and increased youth populations in emerging economies, profoundly affect the labor dynamics, consumption patterns, and strategic investments of food systems. In such contexts, blockchain can enhance intergenerational knowledge, while providing transparent land lease and clear records of sustainability performance. Blockchain’s records help labor mobility, skills qualifications, and trust in youth engagement in agriculture [2].

Furthermore, blockchain tools provide reasonable and safer access to finance, training, and entrepreneurship opportunities, eliminating exclusion, while leading innovation in underserved areas. In regions facing economic disparities, BCT can provide opportunities for effective labor matching and social protection [55]. However, these activities require public and private effort to ensure digital literacy, affordable and fair access to relevant infrastructure and platforms, and governance models.

#### 3.2.6. Shifting Health Challenges

Today, food systems are facing new health challenges, such as the rise of non-communicable diseases (NCDs), including obesity and diabetes, micronutrient shortages, emerging foodborne diseases, and consumer demand for safer and healthier foods. End-to-end traceability provided by blockchain safeguards that the whole food supply chain is recorded and immutable. This helps in rapidly identifying and recalling contaminated products during crises, reducing the risks associated with fraud and delayed responses. Transparency in production and distribution also fosters better management of allergen risks, cross-contamination, and food fraud. In addition, for public health authorities and researchers, blockchain data facilitate effective epidemiological monitoring by identifying foods contributing to disease outbreaks. At the same time, blockchain can enable informed choices regarding health, sustainability, and ethical considerations. Finally, interconnecting blockchain records with other digital assets, like health records or wearable devices, can bring about food-tailored dietary interventions, while supporting nutrition programs [56].

On the other hand, some global megatrends present only indirect connections to the adoption of BCT in the agri-food sector. For instance, shifts in work and security examples may generally affect food systems rather than their direct technical transformation. Similarly, rapid urbanization increases competition over land and resources, creating new challenges for food production and distribution. Diversifying education pathways and the growth of new governance structures can affect food system resilience but typically act through broader societal change. The impacts of extending inequalities, and migration, can act as socioeconomic and political drivers shaping agricultural policy, market access, and innovation diffusion [57]. Each of the above trends plays a role in shaping the food system landscape, yet their direct alignment with digitized visibility, decentralized transparency, and blockchain-enabled sustainability remains rather outlying compared to the megatrends.

## 4. Discussion

Interconnection between Megatrends 2030 and scenarios

The forward-looking scenarios of digitization address critical intersections between blockchain technology applications and key megatrends. Below is an analysis of how the megatrends align with the three scenarios: market-led digitization, government-led digitization, and bottom-up driven digitization.

From the European Commission’s full set of 14 megatrends, 6 were selected for in-depth analysis based on three criteria: (i) direct relevance to agri-food systems and food security, (ii) explicit links to digitalization, governance, or environmental sustainability, and (iii) prominence in the stakeholder workshops conducted within the TRUSTyFOOD project. Digitalization and datafication, evolving regulatory landscapes, and growing demand for transparency were consistently ranked by participants as highly influential for blockchain adoption, while megatrends such as climate change and ecological degradation exerted strong but more indirect pressure via regulatory and market responses. Migration dynamics, changing work patterns, and certain security issues were considered important for the broader context and distribution of impacts, but less directly determinative of whether blockchain is adopted in the first place (Table 2).

The megatrend analysis identified several interrelated trends with strong implications for food systems and BCT adoption. These included climate change and ecological degradation, population growth and urbanization, global trade instability, consumer demands for transparency, digitalization and datafication, the rise of decentralized trust mechanisms, and evolving regulatory landscapes. Together, these megatrends reflect a shifting context in which food systems are increasingly required to be not only productive and efficient but also transparent, just, and environmentally sustainable. Blockchain was identified as a potentially enabling infrastructure across several of these trends, particularly in relation to trusted recording, decentralized data management, and accountability mechanisms.

The combined analysis of literature, pilot implementations, and stakeholder inputs indicates that megatrends do not exert uniform influence on blockchain adoption outcomes. Digitalization and datafication, evolving regulatory frameworks, and rising consumer demand for transparency emerge as strong and direct drivers that create concrete incentives to deploy BCT in agri-food supply chains. Climate change and ecological degradation also exert a substantial influence, specifically in contexts where environmental reporting, traceability, and carbon accounting are prioritized. In contrast, megatrends such as migration dynamics, changing work patterns, and certain dimensions of security and education shape the broader socioeconomic context and distributional impacts more indirectly, rather than determining blockchain uptake in a straightforward manner.

The scenarios that were developed during the stakeholder workshops show varied yet plausible blockchain-enabled futures. In particular, the first scenario, titled Efficiency, Transparency, and Resilience, envisions blockchain adoption driven by private sector actors seeking operational efficiency and market responsiveness. In this context, BCT is embedded primarily into business-to-business transactions to rationalize logistics, safeguard traceability, and meet consumer demand for green products. In general, technological innovation and economies of scale advance large agri-food corporations, who invest in blockchain solutions and shape the data ecosystems. While this scenario improves data transparency and carbon accounting, it also raises concerns regarding smallholders’ exclusion, power centralization, and potential greenwashing caused by poor data quality control. The benefits of BCT are concentrated among well-capitalized actors, leading to a food system that is technologically advanced but socially uneven.

Such risks indicate the need for deliberate governance and design choices that integrated blockchain within inclusive and accountable food system transformations. From a policy perspective, targeted financial support, capacity-building programs, and advisory services can help enable smallholders and SMEs to participate in blockchain-based systems. From a technical and institutional design perspective, the promotion of open standards and interoperable architectures, public or multi-stakeholder oversight of consortia, and clear data governance frameworks can decrease power asymmetries. In addition, embedding social safeguards and participatory mechanisms within blockchain initiatives—such as co-design processes involving farmer organizations, consumer groups, and civil-society actors—can mitigate risks of exclusion, enhance trust, and ensure that digital innovation supports, rather than undermines, equity in agri-food-system equity.

To move beyond risk identification and provide actionable guidance, the analysis was synthesized into a structured risk mitigation framework. Table 3 maps the main governance and design risks associated with blockchain adoption in agri-food systems to corresponding mitigation mechanisms and policy or design responses. This mapping translates the empirical and scenario-based findings into concrete interventions that can be applied by policymakers, regulators, and system designers to reduce exclusion, limit power asymmetries, and enhance accountability in blockchain-enabled food systems.

The second scenario, Compliance and Accountability, is centered around a policy-driven transformation in which governments adopt BCT to enforce environmental and social regulations. This scenario associates BCT adoption with policy instruments, including the EU Green Deal, Corporate Sustainability Reporting Directive (CSRD), and the EU Deforestation Regulation (EUDR). National authorities establish common standards and terminology, promoting interoperability of national and regional blockchain systems. BCT is used as a backend infrastructure to achieve data verification, regulatory compliance, as well as monitoring of sustainability indicators. In parallel, governments implement measures to support smallholder adoption through subsidies, education, and training. This scenario improves regulatory coherence and public trust, but also indicates potential risks related to surveillance, loss of autonomy, and increased administrative burdens, especially for resource-constrained actors. On the other hand, the third scenario, Ecologically Integrated and Place-Based, illustrates a bottom-up transformation led by local communities, small-scale producers, and cooperatives. In this case, BCT is adopted through participatory and decentralized initiatives alongside circular economy and food independence principles. Applications include Participatory Guarantee Systems (PGS), local crowdfunding for sustainable farming, community-managed resource governance, and direct consumer involvement. Furthermore, BCT is also used for citizens’ empowerment and enhancing autonomy over data and decision-making. This scenario shows a strong alignment between environmental sustainability, social equity, and advanced digital technologies. Yet, the localized and fragmented nature of technological innovation brings challenges in scalability, standardization, and interoperability. Several communities lack the digital infrastructure, financial funds, and/or technical support that are necessary to maintain such systems over time, and the risk of marginalization remains if these models are not institutionally supported.

Across all three scenarios, two primary applications of blockchain emerged: transparency and visibility in supply chains, and accounting for environmental externalities, such as carbon emissions. However, the purpose, governance, and beneficiaries of these applications vary substantially. While the first scenario sees markets as the primary agents of transformation, the second emphasizes the regulatory state, and the third prioritizes community-led initiatives. The scenarios also differ in terms of their treatment of values, including decentralization, equity, trust, and inclusiveness. All the selected scenarios underline that the outcomes of blockchain deployment in food systems are not predetermined. Blockchain can lead to vastly different impacts depending on how it is integrated within broader systems of governance, power, and participation.

The stakeholder workshops resulted in three distinct scenario descriptions that differ primarily in their underlying governance logic and in the actors who benefit most from blockchain deployment. More specifically, the “Market-Led Digitization” scenario is driven by large private-sector actors, prioritizing efficiency gains and market responsiveness. The “Compliance and Accountability” scenario reflects a government-led configuration in which public authorities deploy BCT to enforce regulatory and sustainability objectives. In contrast, the “Ecologically Integrated and Place-Based” scenario follows a bottom-up logic, driven by local communities, small-scale producers, and civil-society organizations that integrated blockchain within territorial and circular economy logics. To enhance conceptual clarity, the scenario labels have been harmonized throughout the manuscript to explicitly reflect these distinct governance rationales.

### 4.1. Market-Led Digitization

This scenario envisions a food system where the private sector drives digitization, with blockchain and digital tools primarily being used for market efficiency and meeting consumer demands. This scenario aligns with the megatrend of rapid technological change. Blockchain, IoT, and advanced logistics are at the core, enabling transparency, traceability, and market-driven innovations. While the scenario focuses on market efficiency and sustainability initiatives, these efforts might be more about profit and differentiation than addressing resource scarcity in a transformative way. Smaller actors could be excluded, leaving resource management fragmented.

Although sustainability is a market demand, the risk of greenwashing in the scenario suggests that true environmental impact might be minimal. The megatrend of climate change may be addressed superficially, without deep structural changes. This scenario considers the environmental megatrend only to the extent that it serves market goals. Corporations respond to regulations and consumer preferences for sustainability, but the focus on profits means that environmental degradation may not be fully addressed in a transformative way.

In addition, consumer demand for transparency, sustainability, and traceability is a significant driver in this scenario. Market-led digitization responds to this trend by using blockchain to offer consumers more information on their food’s origins and ecological impact, though primarily to maximize profits. Companies may also focus on transparency in nutritional labeling, catering to health-conscious consumers. However, without systemic change, these solutions could benefit only niche markets and fail to address widespread dietary-related diseases or malnutrition.

### 4.2. Government-Led Digitization

This scenario presents a vision where governments take the lead in driving digitization, using blockchain to enforce sustainability and public accountability in the food system. This scenario embraces technology, but with an emphasis on public oversight and standardization. Blockchain is a tool for regulatory enforcement, aligning well with technological megatrends but with a different emphasis than the private sector.

This scenario also aligns with efforts to address climate change through government-enforced sustainability goals. The use of technology for traceability and public transparency could help achieve climate and environmental goals, echoing the global call for action under this megatrend. The scenario fully embraces the megatrend, with climate action at the center of government policies, ensuring that the food system contributes to reducing greenhouse gas emissions and environmental degradation.

Furthermore, governments would likely focus on sustainable resource use and equitable access, addressing the megatrend of resource scarcity by ensuring that blockchain technology is used to support sustainable farming practices and optimize resource distribution. By mandating sustainability measures, governments work to mitigate the impacts of resource depletion. Public funding and educational initiatives also provide equal access to digital tools, bridging gaps across different demographic groups, including aging farmers and youth in agriculture. Finally, governments enforce food safety and nutrition regulations, using blockchain to ensure compliance and transparency in addressing public health concerns, such as foodborne illnesses and non-communicable diseases.

### 4.3. Bottom-Up-Driven Digitization

This scenario emphasizes grassroots initiatives, where local communities, cooperatives, and small farmers drive the adoption of blockchain technology to increase local autonomy, resilience, and sustainability. Grassroots-driven blockchain adoption enables localized solutions for transparency and resource tracking, fostering innovation tailored to community needs. This scenario directly addresses resource scarcity by promoting local and decentralized control over agricultural resources. The focus on agroecology and regenerative systems supports sustainable resource management, aligning well with the megatrend of addressing environmental limits.

Besides, bottom-up approaches to agroecology and circular economy practices are aligned with the need to combat climate change and environmental degradation. Communities’ control over resources and food systems could lead to more sustainable practices and resilience against climate shocks. Community-driven digitization enables tailored approaches to health concerns, such as tracking local nutritional needs and empowering consumers to access healthier food choices directly from producers.

## 5. Conclusions

Blockchain technology offers significant potential to contribute to the transformation of food systems by enabling new forms of transparency, accountability, and decentralized governance. Yet, realizing this potential depends on navigating complex value tensions, mitigating exclusion risks, and addressing implementation with broader societal goals. All three scenarios analyzed in the study provide insights into how BCT could evolve in the food sector under different configurations of policy and innovation. This analysis stresses the importance of participatory governance, value-based design, and institutional support in supporting blockchain adoption toward equitable and sustainable outcomes. Policymakers, food system actors, and technologists must co-design frameworks that ensure data integrity, promote interoperability, and empower diverse actors, particularly those who are often marginalized in technological transitions.

The foresight approach applied in this study demonstrates the value of participatory scenario development in clarifying the stakes of technological innovation in complex sociotechnical systems. As food systems continue to digitalize, the need for anticipatory, inclusive, and reflexive governance will only grow. BCT, if implemented inclusively, can be one of the enabling tools in this transformation, supporting pathways that are not only efficient and transparent but also just, inclusive, and ecologically sound.

Building on these findings, several directions for future research emerge. Empirical validation of the scenarios developed in this study—through in-depth case studies, policy pilots, or comparative analyses across regions—would enable assessment of how different governance logics shape real-world blockchain outcomes. In addition, there is a need for systematic, long-term evaluations of blockchain implementations in diverse agri-food contexts, including quantitative assessment of environmental and social impacts beyond traceability performance alone. Finally, comparative studies of alternative governance models—such as corporate consortia, public-led infrastructures, and community-based systems—could clarify how benefits and risks are distributed across actors and inform the design of more inclusive and accountable digital food system infrastructures.

## Figures and Tables

**Table 1:** Reported benefits of blockchain adoption in agri-food systems, evidence type, and example metrics.

Benefit	Type of Evidence	Example Metrics/Indicators	Illustrative Sources
Faster audit and certification processes	Literature and pilot cases	Reduction in audit time (days/weeks); fewer manual verification steps	Case studies; EU pilots
Improved recall performance	Pilot cases	Time to identify affected batches; recall response time	Food safety pilots
Enhanced traceability granularity	Literature and pilot cases	Batch-level vs. item-level traceability; data completeness	Academic studies
Reduced administrative burden	Pilot cases	Reduction in paperwork; number of automated transactions	Supply chain pilots
Improved data integrity	Literature	Tamper-resistant; immutability features	Conceptual and empirical studies
Increased consumer trust	Literature	Survey-based trust indicators; transparency perception	Consumer studies

**Table 2:** Relative influence of selected megatrends on blockchain adoption in agri-food systems.

Megatrend	Relevance to Agri-Food	Link to Digitalization and Governance	Stakeholder Salience	Influence on Blockchain Adoption
Digitalization and datafication	High	High	High	Strong/Direct
Regulatory evolution and transparency demand	High	High	High	Strong/Direct
Climate change and environmental degradation	High	Medium–High	High	Strong/Direct
Resource scarcity	Medium–High	Medium	Medium	Moderate/Direct
Demographic change	Medium	Low–Medium	Medium	Indirect/Contextual
Health and food safety challenges	Medium	Medium	Medium	Indirect/Contextual

**Table 3:** Risk mitigation map for blockchain adoption in agri-food systems.

Identified Risk	Description	Governance Mechanism	Policy/Design Response
Exclusion of smallholders	High entry costs, technical barriers, digital divide	Multi-stakeholder governance; public oversight	Targeted financial support; capacity-building programs; advisory services
Power concentration	Control by dominant platforms or consortia	Transparency and accountability requirements	Open and interoperable standards; anti-lock-in regulation
Data appropriation	Unequal control over data access and reuse	Data governance frameworks	Clear rules on data ownership, access rights, and consent
Vendor lock-in	Dependence on proprietary blockchain solutions	Standardization bodies; public procurement rules	Interoperable architectures; open-source components
Reduced trust	Lack of legitimacy or social acceptance	Participatory governance	Co-design with farmers, consumers, and civil-society organizations

## Data Availability

The original contributions presented in this study are included in the article. Further inquiries can be directed to the corresponding author.

## Abbreviations

The following abbreviations are used in this manuscript:

BCT	Blockchain Technology
NCDs	Non-Communicable Diseases
CSRD	Corporate Sustainability Reporting Directive
EUDR	EU Deforestation Regulation
PGS	Participatory Guarantee Systems
IoT	Internet of Things
